# O-GlcNAcylation links oncogenic signals and cancer epigenetics

**DOI:** 10.1007/s12672-021-00450-5

**Published:** 2021-11-24

**Authors:** Lidong Sun, Suli Lv, Tanjing Song

**Affiliations:** Department of Biochemistry and Molecular Biology, School of Basic Medicine, Tongji Medical College, Huazhong University of Science and Technology, 13 Hangkong Road, Wuhan, 430030 China

**Keywords:** O-GlcNAcylation, Hexoamine biosynthesis pathway, OGT, OGA, Histone, Chromatin, Epigenetics

## Abstract

Prevalent dysregulation of epigenetic modifications plays a pivotal role in cancer. Targeting epigenetic abnormality is a new strategy for cancer therapy. Understanding how conventional oncogenic factors cause epigenetic abnormality is of great basic and translational value. O-GlcNAcylation is a protein modification which affects physiology and pathophysiology. In mammals, O-GlcNAcylation is catalyzed by one single enzyme OGT and removed by one single enzyme OGA. O-GlcNAcylation is affected by the availability of the donor, UDP-GlcNAc, generated by the serial enzymatic reactions in the hexoamine biogenesis pathway (HBP). O-GlcNAcylation regulates a wide spectrum of substrates including many proteins involved in epigenetic modification. Like epigenetic modifications, abnormality of O-GlcNAcylation is also common in cancer. Studies have revealed substantial impact on HBP enzymes and OGT/OGA by oncogenic signals. In this review, we will first summarize how oncogenic signals regulate HBP enzymes, OGT and OGA in cancer. We will then integrate this knowledge with the up to date understanding how O-GlcNAcylation regulates epigenetic machinery. With this, we propose a signal axis from oncogenic signals through O-GlcNAcylation dysregulation to epigenetic abnormality in cancer. Further elucidation of this axis will not only advance our understanding of cancer biology but also provide new revenues towards cancer therapy.

## Introduction

### O-GlcNAcylation and hexoamine biosynthesis pathway

O-GlcNAcylation is a reversible mono-saccharide modification on protein serine or threonine residues with UDP-GlcNAc as the donor. Unlike polysaccharide-modifications occurring in ER and Golgi apparatus, O-GlcNAcylation takes place mainly in cytosol and nucleus. O-GlcNAcylation is added by o-GlcNAcyl transferase (OGT) and removed by O-GlcNAcase (OGA). O-GlcNAcylation is conserved in metazoan and plants. In mammalian cells, OGT is encoded by a single copy X-linked gene, while in plants, there are two homologs [1].

**Fig. 1:**
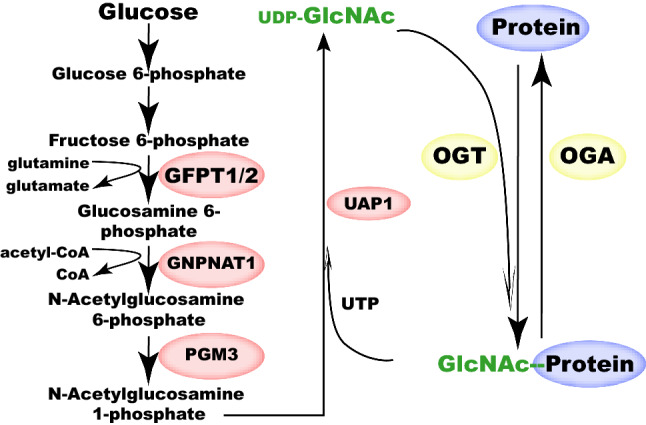
The HBP pathway and Protein O-GlcNAcylation. GFPT1/2, GNPNAT1, PGM3, UAP1 in the HBP pathway generates UDP-GlcNAc. OGT can then O-GlcNAcylate protein substrates with UDP-GlcNAc as donor. O-GlcNAcylation has pleiotropic effects on substrates and can be removed by OGA

UDP-GlcNAc is generated by a serial of enzymatic reactions in Hexoamine Biosynthetic Pathway (HBP). Although often referred to as a sub-pathway of glucose metabolism, HBP integrates inputs from energy (ATP), glutamate metabolism (providing amine), fatty acid metabolism (providing acetyl-CoA) and nucleotide metabolism (providing UTP) as well (Fig. 1). HBP enzymesinclude GFPT1/GFPT2, GNPNAT1, PGM3 and UAP1 (Fig. 1). GFPT1/GFPT2 is usually considered rate-limiting in this pathway. GFPT1 is ubiquitously expressed while GFPT2 is highly expressed in the central nervous system [2, 3].

Proteomic studies have found thousands of proteins are O-GlcNAcylated. O-GlcNAcylation has pleiotropic effects on substrates, including changes of localization, stability, activity and protein–protein interaction. O-GlcNAcylation essentially affects every aspect of cell biology, including metabolism homeostasis, cell signaling, DNA damage repair, gene expression, protein translation and quality control. Consistently, knocking-out any of OGT [4], OGA [5, 6], GFPT1 [7], GNPNAT1 [8] and PGM3 [9] causes embryonic or neonatal lethality. Dysregulation of O-GlcNAcylation is associated with many diseases including diabetes, neurodegenerative diseases and cancer. Abnormal O-GlcNAcylation contributes to oncogenesis and cancer progression through regulating signal transduction, gene expression and metabolism [10–13].

### Epigenetics and its dysregulation in cancer

Like O-GlcNAcylation, dysregulation of epigenetic mechanisms also contributes to cancer [14, 15]. Epigenetics refers to mechanisms of inheritable changes in gene expression which are not dependent on change in DNA sequence. Histone modifications, DNA methylation and chromatin remodeling are among the most studied epigenetic mechanisms [16]. Besides, non-coding RNA and histone chaperone also contribute significantly to gene transcription. Histone modifications including acetylation/ubiquitination on lysine, methylation on lysine/arginine, phosphorylation on serine/threonine/tyrosine and O-GlcNAcylation on serine/threonine have important roles in chromatin biology. All these modifications are installed by dedicated enzymes, the “writers”. Specifically, histone lysine acetylation is catalyzed by histone acetyltransferases. Lysine ubiquitination is catalyzed by site-specific E3 ubiquitin ligases with the help of E2. Lysine methylation is catalyzed by a group of lysine methyltransferases while arginine methylation is catalyzed by a group of protein arginine methyltransferases [17]. Phosphorylation are catalyzed by site-specific kinases. Histone O-GlcNAcylation is catalyzed by OGT. These modifications are reversible and can be removed by counter-acting enzymes including deacetylases, demethylases, deubiquitinases, phosphotases and OGA, respectively. DNA methylation can be oxidized by TET1/2/3 and then removed by nucleotide glycosidase. Enzymes removing epigenetic modifications are referred to as “eraser”. Several different mechanisms may be employed to translate the epigenetic modifications into biological functions. One mechanism is that the modification can directly facilitate or obstruct the binding of a functional protein to chromatin including transcription factors, RNAP. Another mechanism is a modification may directly change the higher structure of the chromatin. A third mechanism is that the modification serves as a docking site for a specific recognizing protein. There are many dedicated protein domains specifically recognizing the epigenetic modifications, especially for histone methylation [18], acetylation, phosphorylation [19] and DNA methylation [20]. These recognizing proteins are referred to as “readers”. In reality, epigenetic writers, erasers and readers very often function within a multicomponent complex. Many well-studied protein complexes have more than one enzymatic activities or possess both catalytic activity and reader activity. Auxiliary subunits in these protein complexes may modulate the activity or the genome targeting of the writer/eraser/reader. In some cases, without auxiliary subunits, the catalytic subunits, for example histone methyltransferases EZH2 and MLL, have no detectible activity towards histone or nucleosome at all [21–23]. In some cases, one catalytic subunit may give rise to functionally different protein complexes in which auxiliary subunits render different substrate specificity or genomic targeting. Another common theme in epigenetics is one epigenetic modification may either facilitate or interfere with another modification. Such cross-talking between epigenetic modifications can occur in the same histone molecule or across different histone molecules, which often involves the first modification directly or indirectly engaging the enzyme catalyzing the second modification. Epigenetics affects every aspect of the genome including DNA replication, repair and transcription. Epigenetics plays pivotal roles in development and cancer. Abnormality in the writers, readers, erasers of epigenetic modifications contributes to cancer [24, 25].

An important question regarding both epigenetics and o-GlcNAcylation is what cause their abnormality in cancer. For epigenetic machinery, besides genetic changes in their-coding genes, they are regulated by many of the well-known oncogenic signals [26]. For O-GlcNAcylation, besides the regulation from metabolic flux [12], HBP enzymes, OGT and OGA are regulated by cancer-related pathways as well. Next, we will first review how HBP enzymes, OGT and OGA is regulated by intracellular and extracellular oncogenic factors. We will then discuss how o-GlcNAcylation directly regulates epigenetic machinery. With this, we highlight O-GlcNAcylation as a potent mediator between oncogenic signals and cancer epigenetics.

## Signaling pathways regulate HBP enzymes, OGT and OGA

O-GlcNAcylation level is increased in most cancer types [11]. Expression of OGT is also observed in many cancer types, as summarized in Table 1 and elegantly reviewed previously [13].

**Table 1:** Studies showing abnormal O-GlcNAcylation in cancer

Cancer type	Finding	Citation
Pancreas	Excessive O-GlcNAcylation is anti-apoptotic	[27]
Ovary	O-GlcNAcylation, cell migration and changes in E-Catherin level are correlated	[28]
HCC	O-GlcNAcylation is linked with tumor recurrence	[29]
Bladder	Content of OGT/OGA mRNAs helps predicting bladder cancer	[30]
Cholangiocarcinoma	OGT overexpression and aggressiveness are correlated	[31]
Prostate	Overexpression of OGT contributes to invasion, angiogenesis, and metastasis, associates with poor prognosis of patients	[32–34]
Endometrium	Clinicpathologic conditions are correlated with OGT and OGA mRNA expression	[35]
Lung and colon	OGT is overexpressed in cancer compared to adjacent tissues. Treatment with TMG increased cell anchorage-independent growth and invasion in cell lines	[36, 37]
Chronic lymphocytic leukemia	Indolent and aggressive clinical behavior of CLL cells were found to correlate with higher and lower O-GlcNAc levels, respectively	[38]
Thyroid cancer	OGA enzyme activity is increased, cancer has less O-GlcNAcylated proteins	[39]
Breast	Increased OGT level, increased o-GlcNAcylation level enhances the migration/invasion. Higher level of OGT and lower OGA was seen in higher grade of breast cancer tissues	[40, 41]
Esophageal cancer	OGT is overexpressed and promotes O-linked protein glycosylation	[42]

A ensuing and compelling question is how O-GlcNAcylation gets dysregulated in cancer. One important cause is the metabolic abnormality in cancer, which has been reviewed elegantly elsewhere [11, 43–46]. Another potential cause is dysregulation of HBP enzymes and OGT/OGA, which will be elaborated next in this review (Table 2).

**Table 2:** Regulation of HBP enzymes and OGT/OGA by oncogenic signals

Signal	Target	Mode of action	Effect	Citation
mTORC2	GFPT1, GNPNAT1, PGM3	Transcription	Increase expression	[49, 50]
	GFPT1	S243 phosphorylation	Increase protein level	[52]
	OGT	Post-translational	Increase protein level	[53]
GSK3β	OGT	S3, S4 phosphorylation	Increase activity	[54]
KRAS	GFPT1		Increase expression	[55]
HRAS	OGT		Increase expression	[56]
AMPK	OGT		Increase mRNA	[63]
	OGT	T444 phosphorylation	Decrease interaction with chromatin	[61]
	OGT	T444 phosphorylation	Change substrate preference, increase nuclear localization	[62]
	GFPT1	S243 phosphorylation	Change activity	[64–66]
PKA	OGT	Post-translation	Decrease activity	[68]
	GFPT1	S205 phosphorylation	Affect activity	[69–71, 73]
CAMKII	OGT	S20 phosphorylation	Increase activity	[80]
CAMKIV	OGT	Phosphorylation	Increase activity	[81]
CHK1	OGT	S20 phosphorylation	Stabilize OGT, OGT localization to midbody	[83]
RB1-E2F1	OGT	Transcription	Decrease expression	[85]
FOXA2	GFPT1	Transcription	Increase expression	[93]
NR4A1	GFPT2	Transcription	Increase expression	[95]
AIbZIP	GFPT1		Increase expression	[97]
ATF4	GFPT1	Transcription	Increase expression	[91]
NF-κB	GFPT2	Transcription	Increase expression	[86]
NRF2	OGT	Transcription	Increase expression	[87]
HNF1	OGT	Transcription	Increase expression	[100]
SP1	GFPT1	Transcription	Increase expression	[101]
CREB	OGT	Transcription	Affect OGT mRNA level	[98]
C/EBPβ	OGT	Transcription	Expression	[57]
SIRT6	GFPT2	Transcription	Decrease expression	[102]
mSIN3A-HDAC1	OGA	Transcription	Decrease expression	[103]
microRNA-101	OGT	Post-transcription	Decrease OGT protein level	[107]
miR-15b	OGT	Post-transcription	Decrease OGT protein level	[108]
miRNA-200a/miRNA-200b	OGT	Post-transcription	Decrease OGT protein level	[109]
miR-501-3p	OGT	Post-transcription	Decrease OGT protein level	[110]
miR-619-3p	OGT	Post-transcription	Decrease OGT protein level	[110]
MicroRNA-539	OGA	Post-transcription	Decrease OGA protein level	[111]
LSD2	OGT	Ubiquitination	OGT protein degradation	[112]
XIAP	OGT	Ubiquitination	OGT protein degradation	[113]
β-TrCP1	OGT	Ubiquitination	OGT protein degradation	[114]
BAP1	OGT	Deubiquitination	Promote OGT stability	[116]
TG2	GFPT	PTM	Increase activity	[119]
OGT	OGT	S389 O-GlcNAcylation	Critical for nuclear localization	[121]
UAP1L1	OGT	PPI	Increase activity	[122]
Fatty acid synthase	OGA	PPI	Decrease activity	[123]
TET2/3	OGT	PPI	Promotes chromatin targeting	[124, 125]
TET1/2/3	OGT	PPI	Promote protein stability	[126]
P38	OGT	PPI	Bridges targeting	[63]
PI3K	OGT		Increase expression	[127]
PI3K	OGT		Increase localization to plasma membrane	[128]
Insulin Receptor	OGT	Phosphorylation	Increase expression, promotes plasma membrane localization	[129]
EGF	GFPT1		Increase expression	[131]
hypoxia	Gfpt2		Increase expression	[132]
hypoxia	OGT		Decrease expression	[114]
HPV-E6	OGT		Increase expression	[133]
HTLV-1 Tax	OGA		Decrease OGA mRNA level and inhibit OGA activity	[134]

Many signaling pathways can regulate HBP enzymes and OGT/OGA at transcriptional, post-transcriptional and post-translational levels. As discussed below, most of these signal pathways are either most notable oncogenic pathways or functionally implicated in cancer. Besides intracellular signaling proteins, oncogenic signals from tumor microenvironment and virus oncoproteins can regulate O-GlcNAcylation through HBP enzymes and OGT/OGA. Consequently, expression, activity, localization, substrate targeting, substrate preference of HBP enzymes and OGT/OGA can change, giving rise to pleiotropic downstream effects.

### PI3K/AKT/mTOR pathway

PI3K/AKT/MTOR pathway is one of the most commonly activated pathways in cancer. This pathway can regulate multiple HBP enzymes and OGT/OGA through different mechanisms.

MTOR can constitute two kinase complexes, mTORC1 and mTORC2, both of which play important roles in cancer. Disrupting mTORC2 function by knocking-out mTORC2 component SIN1 decreases UDP-GlcNAC level and O-GlcNAcylation level in mouse embryonic fibroblast (MEF) cell, which is concordant with decreased GFPT1 and UAP1 level [47]. Mechanistically, SIN1-KO decreases XBP1 mRNA level and XBP1s protein level in the nucleus [47]. XBP1 is a master transcription factor in unfolded protein response and production of the active isoform, XBP1s, due to endonuclease-dependent pre-mRNA processing plays an important role in ER stress. XBP1s is often increased in cancer and promotes cancer progression in many cancer types [48]. Importantly, XBP1s functions as a direct transcriptional activator of GFPT1 [49, 50]. In mouse cell, XBP1s directly binds to the promoter of GFPT1, GNPNAT1 and PGM3[49]. Consistently, overexpressing XBP1s in human cervical cancer HeLa cells upregulates expression of HBP enzymes such as PGM3 and UAP1 [51]. In addition to XBP1s, mTORC2 maintains α-Ketoglutarate level in the cell, which is also important for GFPT1 level [47].

Besides regulating GFPT1 transcription, mTORC2 can also bind and phosphorylate GFPT1 directly at S243 [52], which increases GFPT1 protein level [52]. PTEN is a tumor suppressor and a major endogenous negative regulator of PI3K/AKT/mTOR signal. In cancer, PTEN gene often carries loss-of-function mutation. In PTEN-null mouse lymphoma model where mTORC2 activity is augmented, GFPT1-S243 phosphorylation level and O-GlcNAcylation level are also increased [52]. Pertinent to its regulation by phosphorylation, GFPT was shown to interact with PPP2R2A. Knockdown of PPP2R2A increases phosphorylation of GFPT2, indicating GFPT phosphorylation could be under dynamic control by both kinases and phosphotases [58-Li, X.-2018-Sheng Wu Gong Cheng Xue Bao].

mTOR can also increase expression of OGT. In breast cancer cells, treatment with PI3K or mTOR inhibitors decreases O-GlcNAc level, which is dependent on C-MYC [53]. C-MYC itself is a transcription factor overexpressed or activated in many tumors. C-MYC does not seem to directly regulate OGT transcription as the increase in OGT protein level is not accompanied by change in OGT mRNA. Instead, C-MYC increases OGT through HSP90A, an important molecular chaperone and a transcriptional target of C-MYC. In vivo, OGT and O-GlcNAcylation levels are also increased in mouse mammary tumor induced by transgenic C-MYC [53].

GSK3β is another important protein kinase which can function downstream of PI3K/AKT. GSK3β can directly phosphorylate OGT at Ser3 and Ser4, which modestly increases OGT enzymatic activity [54]. In vitro results indicate phosphorylation at these two sites may enhance the effect of potential phosphorylation at other sites by GSK3. GSK3-mediated OGT phosphorylation plays important roles in Circadian clock [54].

### RAS-MAPK

RAS-MAPK pathway is another commonly over-activated signal pathway in cancer. O-GlcNAcylation is proven to be an important mediator of its biological effects. KRAS is mutated and activated in about 25% of all cancer and 90% of pancreatic ductal adenocarcinoma (PDA). Activation of KRAS, the most important oncogene in PDA, increases HBP flux by upregulating GFPT1 mRNA and protein level [55]. Consistently, inhibiting KRAS decreases cellular O-GlcNAcylation level. KRAS upregulates GFPT1 through MAPK pathway, as MEK inhibitor AZD8330 treatment decreases GFPT1 mRNA level [55]. Importantly, knocking-down GFPT1 decreases KRAS tumor growth in vitro and in mice model [55]. HRAS is another member of the RAS family oncogenes. Overexpressing constitutively active H-Ras-V12 in IMR-90 also increases OGT level [56]. Besides, OGA activity is decreased upon inhibiting ERK activity as well, indicating RAS-MAPK pathway regulates both installation and removal of O-GlcNAcylation [57].

### AMP-activated protein kinase (AMPK)

Protein kinase AMPK is a major signal hub sensing cellular metabolism and energy status [58, 59]. As a hetero-trimer, each AMPK contains one AMPK-β and one AMPK-γ subunit in addition to the catalytic AMPK-α subunit. Relation between AMPK and cancer has been under intense study. It seems AMPK has a tumor suppressive role before tumorigenesis but may benefit tumor cell survival during cancer progression. There are two AMPK-α subunits in the cell, which may function differently in cancer [60].

Several studies have linked regulating cellular O-GlcNAcylation to AMPK function. AMPK regulates OGT through different mechanisms. AMPK can directly phosphorylate OGT at Thr-444 [61, 62]. While Thr-444 phosphorylation does not seem to change OGT enzymatic activity directly, it decreases OGT interaction with chromatin and hence histone H2B O-GlcNAcylation [61]. Meanwhile, Thr-444 phosphorylation changes OGT substrate preference globally [62]. Besides, AMPK may affect OGT subcellular localization. In myotubes, AMPK activity correlates with increased OGT localization to the nucleus [62]. In addition, AMPK may increase OGT mRNA when it is activated by glucose starvation [63].

Besides OGT, GFPT1 is also directly regulated by AMPK. AMPK can regulate GFPT1 activity by phosphorylating GFPT1-S243, the site paradoxically also phosphorylated by mTORC2 [52]. Earlier in vitro study suggests the S243 phospho-mimic S243E mutant has increased activity [64]. However, after activating AMPK with 2-deoxy-glucose (2-DG) treatment, GFPT enzymatic activity in the cell lysates is decreased [65]. Activity of GFPT1 immunoprecipitated from AMPK-activated cells is decreased by about 30% [66]. In line with decreased GFPT1 activity, AMPK activation decreases overall O-GlcNAcylation level in endothelial cells [66].

### Protein kinase A (PKA)

PKA is a protein kinase and a major effector of the second messenger cAMP. PKA can phosphorylate many downstream protein substrates including other protein kinases and transcription factors. PKA can either promote or suppress tumor depending on the specific cancer type and context [67].

PKA regulates cellular O-GlcNAcylation through multiple mechanisms. PKA can decrease OGT activity through directly phosphorylating Ser-371 of protein URI, a co-chaperone of OGT. URI, PP1γ and OGT together form a complex, in which OGT is active. Upon URI phosphorylation by PKA, PP1γ will be released from this complex, causing decreased OGT activity. Such mechanism is important foradaption to nutrient scarcity by cancer cell when PKA is activated under such condition [68].

PKA also regulates HBP enzymes. PKA can directly phosphorylate GFAT1 at Ser205 and Ser235 [69–71]. While phosphorylation at S235 does not seem to affect GFPT1 activity [70, 72], phosphorylation at S205, which is also conserved in GFPT2 (Ser202 in GFPT2), inhibits activity of GFPT1 [70]. Consistently, in normal rat cell, activation of PKA decreases o-GlcNAcylation. Paradoxically, S202 phosphorylation was reported to increase the activity of GFPT2 [71]. Considering the different expression pattern of GFPT1 and GFPT2, such opposite regulation was suggested to render tissue-specific effects on O-GlcNAcylation by PKA [71]. However, further complicating the situation, GFPT activity increases in rat muscle cells where GFPT1 is supposedly the major form under conditions where PKA is activated, [69]. Recently, with a phospho-mimic S205D mutant, a study reconciles such paradox and shows while phosphorylation of S205 inhibits GFTP1 activity, it also eliminates the feedback inhibition by O-GlcNAc [73].

### Calcium signal

Calcium signal plays an important role in many aspects of cell function. Dysregulation of calcium signaling is involved in cancer [74, 75]. Ca^2+^/calmodulin-dependent protein kinases (CaMK) are a group of important mediators of calcium signal. Their dysregulation is functionally implicated in many cancer types. For example, CaMKII plays an important role in various cancers, such as prostate cancer, liver cancer and neuroblastomas [76–78].

Studies have revealed connection between CaMK and cellular O-GlcNAcylation. CaMKII inhibitor KN93 decreases cellular o-GlcNAcylation level [79]. It was identified later CaMKII could phosphorylate OGT at Ser20, increasing OGT activity and cellular O-GlcNAcylation [80]. Besides, CAMKIV can also phosphorylate OGT and increase its activity to about two-fold, although the targeted site/sites are not known [81].

### Cell cycle-related pathways: CHK1, AURORA-B, RB1-E2F1

CHK1 is a protein kinase important for proper mitosis which can be activated by DNA-damage. Inhibiting CHK1 is an intriguing method to treat cancer, either single-agent or in combination with other drugs [82]. CHK1 functions partly through OGT. CHK1 interacts with OGT, phosphorylates OGT-S20 (the same site phosphorylated by CAMKII) which stabilizes OGT. CHK1-phosphorylated OGT can localize to midbody during mitosis and wherein its activity is important for mitosis [83].

AURORA-B is another protein kinase pivotal for cell division. Also at the midbody, OGT interacts with AURORA-B and protein phosphatase 1. Although it is not known whether Aurora B directly phosphorylates OGT, inhibiting Aurora B abolishes OGT localization to the midbody [84].

RB1 is a well-established tumor-suppressor gene, whose inactivation is seen in many cancer types. RB1 forms a complex with E2F1 and inhibits E2F1-promoted cell proliferation. Interestingly, protein O-GlcNAcylation is a downstream effector of RB1-E2F1. Knocking-out E2F1 decreases cellular O-GlcNAcylation level [85]. Mechanistically, in the presence of RB1, E2F1 can bind to OGT and OGA promoters and decrease their transcription while loss of RB1 increases OGT and OGA mRNA and protein level [85].

### Transcription factors

Many signal pathways regulate gene expression through transcription factors. Besides a few transcription factors already alluded above, many others can regulate the expression of HBP enzymes and OGT/OGA.

#### NF-κB

NF-kB is a master transcription factor involved in many cancer types. NF-kB upregulates transcription of GFPT2, which contributes to lung cancer cell migration and invasion [86].

#### NRF2

NRF2 is a transcription factor most notably known for regulating an important pathway that renders protection from oxidative stress. Under normal condition, NRF2 protein level is under the control of CULLIN3-KEAP1 E3 ligase. NRF2 directly promotes OGT transcription in macrophage. NRF2 knock-out leads to decreased global o-GlcNACylation [87]. Consistently, knocking-out CULLIN3 also increases global O-GlcNAcylation. NRF2 overexpression or KEAP1 loss of function is observed in multiple cancers including hepatoculular carcinoma (HCC) [88]. NRF2 directly promoting OGT is consistent with OGT overexpression in HCC.

#### ATF4

ATF4, a transcription factor often activated by the PERK/GCN2-eIF2α stress-response pathway, is important for tumor cell proliferation and survival [89, 90]. ATF4 can increase GFAT1 transcription and cellular O-GlcNAcylation [91].

#### FOXA2

Transcription factor FOXA2 is functional in different cancer types [92]. A recent study suggests FOXA2 directly activate GFPT1 transcription, which plays a role in HCC sensitivity to doxorubicin [93].

#### NR4A1

NR4A1, an orphan nuclear receptor, functions as a transcription factor. It can regulate many different cellular processes and is implicated in many types of malignancies [94]. NR4A1 increases the mRNA level of GFPT2 but not GFPT1 in retinal cell, leading to increased O-GlcNAcylation [95].

#### AIbZIP

AlbZIP is a bZIP-family transcription factor overexpressed in prostate cancer [96]. AlbZIP expression is induced by androgen receptor [96]. The mechanism of AlbZIP function in cancer is not well-understood. AlbZIP increases GFPT1 mRNA level, although whether it directly targets GFPT1 is not known [97].

#### CREB

CREB is another multifunction transcription factor. CREB can be phosphorylated and activated by many protein kinases. Hyper-activation of CREB, frequently observed in cancer, supports tumor initiation and progression. Knocking-down CREB increases OGT mRNA level [98].

#### HNF1

HNF1 is a transcription factor implicated in several cancer types [99]. HNF1 was reported to have a modest effect on OGT mRNA level [100].

#### SP1

Albeit widely-viewed as a basic transcription factor for house-keeping genes, SP1 also regulates expression of oncogenes and tumor suppressor genes. Overexpression of SP1 is associated with poor prognosis in many cancer types and SP1 is subject to regulation by various post-translational modifications. It was shown Sp1 could regulate expression of GFPT1 [101].

#### C/EBPβ

Transcription factor C/EBPβ can upregulate OGT transcription through cooperation with histone acetyltransferase P300 [57].

Above transcription factors are subject to regulation of many upstream signal pathways. They likely function as important mediators between oncogenic signals and changes in HBP enzymes as well as OGT/OGA.

Besides transcription factors, epigenetics factors can also regulate the expression of HBP enzymes and OGT/OGA. For example, SIRT6 downregulates the transcription of GFPT2, which plays a role in lung cancer cell migration and invasion induced by NF-kB [86]. Besides, PRC2 complex is required to maintain OGT protein level [102]. mSIN3A-HDAC1 complex can repress the transcription of OGA [103]. SIRT1 can repress the expression of OGT through CREB deacetylation [98].

### Splicing

Besides transcriptional and posttranslational regulation, expression of OGT can also be regulated at splicing stage. OGT splicing variants ncOGT, sOGT and mOGT, have different localization tendency [104, 105]. In addition, splicing can also regulate OGT protein level as intron retention can decrease levels of mature OGT mRNA. Interestingly, intron retention in OGT mRNA is promoted by cellular O-GlcNACylation level, representing an intrinsic mechanism for O-GlcNAcylation homeostasis [106]. But what transactivation factors may regulate OGT splicing and how OGT splicing is regulated by oncogenic signals remain to be revealed.

### miRNA

miRNA represents another important mechanism for regulation of gene expression. Study on miRNA has greatly expanded our understanding of human genome. Many miRNAs are involved in cancer. Studies have identified functional links between miRNA and cellular O-GlcNAcylation. miR101 is tumor-suppressive in multiple cancer types. microRNA-101 can post-transcriptionally inhibit OGT expression [107]. In addition, OGT expression is regulated by miR-15b [108], miRNA-200a/miRNA-200b [109], miR-501-3p and miR-619-3p [110]. Besides, miRNA-539 is reported to suppress OGA expression [111]. All these miRNAs are implicated in cancer.

### Ubiquitin–proteasome system

As alluded to above, some signal pathways regulate OGT protein stability. Consistently, OGT can be regulated by ubiquitin–proteasome pathway. LSD2 [112] and XIAP can serve as E3 ligases of OGT [113] and promote its degradation. In addition, E3 ligase β-TrCP1 can also downregulate OGT [114]. All these three E3 ligases are implicated in cancer. OGT also interacts with another E3 ligase, HUWE1, but whether HUWE1 ubiquitinates OGT is not clear [115].

Deubiquitinases remove ubiquitination and counteract the effect of E3 ligases. In this regard, OGT, together with HCF-1, can form a protein complex with deubiquitinase BAP1 [116]. BAP1 can deubiquitinate OGT directly. Consistently, loss of BAP1 decreases OGT protein level and cellular O-GlcNAcylation level [116]. Pertinent to this finding, HCF-1, another component in this protein complex, is also required for stabilizing OGT in the nucleus [117].

### Other posttranslational modifications

Besides phosphorylation and ubiquitination discussed above, other posttranslational modifications can also regulate OGT/OGA. Transglutaminase 2 (TG2) is a multifunction enzyme involved in inflammation and cancer. Overexpression of TG2 in several cancers associates with increased cell survival, invasiveness and poor patient prognosis [118]. TG2 can modify Q328 of GFPT1 and increases its enzymatic activity, increasing UDP-GlcNAc biosynthesis and protein O-GlcNAcylation [119]. In addition, during apoptosis, OGA is cleaved by caspase-3 at a noncanonical S_410_VVD_413_ recognition site. As the N- and C-terminal fragments still associate with each other and remain active [120], so the biological consequences of this cleavage is not yet clear. Interestingly, OGT is also self-modified and S389 O-GlcNAcylation is critical for its nuclear localization [121].

### Protein–protein interaction

Many of the factors discussed above interact with and regulate OGT/OGA through posttranslational modifications. Studies have identified other factors that can regulate OGT/OGA through protein–protein interaction yet without involving posttranslational modification of OGT/OGA. Some of these protein–protein interactions modulate OGT/OGA enzymatic activity. One example is UAP1L1, a protein sharing sequence similarity with UAP1 and increased in liver cancer. Abolishing UAP1L1 decreases hematoma cell proliferation. Interestingly, UAP1L1 increases cellular O-GlcNAcylation although itself has little UAP1 activity. Mechanistically, UAP1L1 interacts with OGT and increases OGT activity [122].

Fatty acid synthase, which is overexpressed in many cancer types, can interact with OGA and inhibit its activity [123].

Some other protein–protein interactions regulate OGT subcellular localization and substrate targeting. For example, TET2 and TET3 can interact with OGT and are necessary for global O-GlcNAcylation. TET2 and TET3 are necessary for OGT targeting to chromatin as well as HCF-1 [124, 125]. Consistently, loss of TET2 decreases global O-GlcNACylation and Histone H2B O-GlcNAcylation [125]. Moreover, TET proteins may promote OGT protein stability [126]. Protein kinase P38 can also bind OGT and recruit it to specific targets [63]. In fact, how OGT, a single enzyme, may target specific substrates has been one of the major puzzles in O-GlcNAcylation research. One prominent mechanism is some proteins can function as adaptor between OGT and its substrates [12].

### Factors from tumor microenvironment

Tumor microenvironment has significant influence on tumor initiation and progression. Factors in tumor microenvironment can also regulate HBP enzymes and OGT/OGA.

Insulin is an important hormone regulating both metabolism and cancer progression. Insulin can increase OGT protein level through PI3K signal [127]. Besides, insulin promotes OGT recruitment to plasma membrane through increased PIP3 generated by PI3K [128]. Furthermore, insulin receptor can bind to OGT and phosphorylate OGT upon insulin stimulation in 3T3-L1 cells, which increases OGT activity and O-GlcNAcylation of downstream substrates [129]. The oncoprotein Src was also shown to phosphorylate OGT in vitro [129].

EGF is an important growth factor promoting tumor growth and ERK is a pivotal downstream effector of EGF. EGF can increase GFPT1 gene transcription [101, 130].

IL-8 promotes cancer stem cell-like properties in colon and lung cancer cells [131]. IL-8 can increase GFPT expression, which contributes to IL-8 function [131].

Hypoxia has a profound impact on tumor growth and invasion. Hypoxia can regulate protein O-GlcNAcylation. Gfpt2 was increased up to nine-fold in mouse pancreatic tumor cells (PK4A) following 15 h of hypoxic treatment [132]. Meanwhile hypoxia was reported to decrease OGT protein level in vascular endothelial cell [114].

### Virus oncoproteins

Physical, chemical and biological factors outside of human body contribute to cancer etiology. Chronic virus infection is a major cause to oncogenesis. Some tumorigenic viruses express well-known oncoproteins. For example, HPV may cause cervical cancer as well as head and neck cancer, where HPV-E6 and E7 proteins play a prominent role. Interestingly, OGT and O-GlcNAcylation levels are higher in HPV-caused cervical neoplasm compared to normal cervix [133]. Overexpressing E6 alone has similar effects. Importantly, inhibiting OGT reverses the transformed phenotype caused by HPV-infection. Mechanistically, HPV infection increases expression of several transcription factors including SP-1, NF-kB and c-Myc. With a luciferase reporter assay, it was shown overexpressing these transcription factors could upregulate OGT promoter activity [133].

Human T-cell lymphotropic virus type 1 (HTLV-1) is associated with adult T-Cell leukemia [134]. HTLV-1 expresses Tax oncoprotein, which plays a pivotal role in T-Cell immortalization and leukemogenesis. Interestingly, HTLV-1 Tax oncoprotein can increase cellular O-GlcNAcylation through two mechanisms. Firstly, Tax can decrease the mRNA level of OGA. Secondly, Tax interacts with OGA-OGT complex and inhibits OGA activity [134].

### Regulation of other enzymes in HBP

Most studies have focused on the regulation of GFPT, OGT and OGA as discussed above. While they are key regulators of O-GlcNAcylation, other enzymes in HBP are also important. Abnormality in these enzymes have also been functionally linked to cancer. For example, PGM3 has been reported to be overexpressed in prostate carcinoma [135]. GNPNAT1 is overexpressed in lung adenocarcinoma patients, which correlates with unfavorable prognosis [136]. GNPNAT1 is increased in prostate cancer compared with normal tissue [137] but GNPNAT1 level decreases as tumors become castration-resistant, indicating stage-specific roles of HBP during prostate cancer progression [137]. We recently showed GNPNAT1 knock-down also decreased DOT1L protein level, similar with GFPT1 knock-down. UAP1 is also overexpressed in prostate cancer [135, 137, 138] and bladder cancer [139]. Knocking-down UAP1 mitigates tumor malignancy [139]. Loss of FBXL17, a F-box protein gene often re-arranged in breast cancer, increases cellular O-GlcNAcylation. Mechanistically, FBXL17 binds to UAP1 and inhibits UAP1 phosphorylation [140]. In summary, regulation of HBP enzymes other than GFPT is also important in cancer.

In the future, novel mechanisms underlying the dysregulation of HBP enzymes and OGT/OGA will likely be identified in cancer as well.

As discussed above, transcription factors and miRNA can connect oncogenic signals and O-GlcNAcylation through modulating expression of HBP enzymes and OGT/OGA. In addition, post-translational modifications and protein–protein interaction represent important mechanisms to modulate the protein stability and activity of HBP enzymes as well as OGT/OGA. Compared with mRNA level change, PTM-based change can presumably provide a more prompt response to stimuli. Besides PTMs already discussed above, PhosphoSitePlus database documents a lot more PTMs on HBP enzymes and OGT/OGA [141]. The mechanisms and functions of these modifications are yet to be uncovered. In summary, studies have identified pleiotropic mechanisms how oncogenic signals may regulate O-GlcNAcylation through HBP enzymes and OGT/OGA. It is reasonble to expect that many more new mechanisms will be identified in the future.

## Regulation of epigenetic machinery by O-GlcNAcylation

HBP and OGT/OGA are under the regulation of many oncogenic signals, which may then function through O-GlcNAcylation of downstream substrates. In the past years, epigenetic machinery have emerged as an important part of the O-GlcNAcylation landscape. Next we will summarize the current knowledge how O-GlcNAcylation can regulate epigenetic mechanisms (Table 3).

**Table 3:** O-GlcNAcylation of epigenetic machinery and effects

Substrate	Type	Site	Function	Citation
H2B, H4, H3	Histone	H2B-S36, H4-S47, T32 of H3-T32/S10	Cell cycle regulation	[152–155]
H2AX	Histone	S139	Facilitate DNA damage repair	[159]
H2A	Histone	S40	Facilitate DNA damage repair	[160, 161]
H2B	Histone	S112	Promote gene transcription and DNA damage repair	[162, 165]
H2A	Histone	T101	Loosen chromatin structure	[166]
UBN1	Histone chaperone	S231	Promotes HIRA integrity and function	[56]
EZH2	Histone lysine methyltransferase	S73, S76, S84 and T313	Promotes EZH2 protein stability	[169, 171]
		S729	Abolish H3K27 di- and tri-methylation activity	[170]
DOT1L	Histone lysine methyltransferase	S1511	Stabilize DOT1L	[175]
MLL5	Histone lysine methyltransferase	S435,T440	Promote MLL5 protein stability	[178]
HCF-1	Multifunction	Multiple	cleave and activate HCF1	[182, 183, 223] [224]
CARM1	Histone arginine methyltransferase	S595, S598, T601, T603	Affect substrate specificity and localization to mitotic chromosome	[155, 187]
NLS3	Histone acetyltransferase-complex	T755	Inhibits NSL3 degradation. Increase histone acetylation	[188, 189]
Mi2-β	Histone Deacetylase-complex		Affect Mi2-β genomic targeting	[191]
MTA1	Histone Deacetylase-complex	S237,S241,S246	Enhance MTA1 association with NuRD complex	[192]
HDAC1	Histone Deacetylase	T114, S145, S263	Increase HDAC1 activity	[195]
HDAC4	Histone Deacetylase	S642	Interferes with S-632 phosphorylation, facilitate HDAC4 cleavage	[197]
SIRT1	Histone Deacetylase	S549	Increase SIRT1 activity	[198]
Bmi-1	Histone Ubiquitination E3 ligase	S255	Stabilize Bmi-1	[202]
RING1B	Histone Ubiquitination E3 ligase	T250, S251, S278	Affect RING1B genomic targeting	[203]
ASXL1	Multifunction	S199	Increase ASXL1 protein stability	[208]
Tet1	DNA demethylation	T535	Increase TET1 protein level	[213]
TET3	DNA demethylation	S1252,S1256,S1263	Promote nuclear export	[215, 216]

### OGT is a polycomb protein and interacts with epigenetic machinery

Polycomb group (PcG) proteins, initially identified in drosophila, is a group of proteins regulating developmental process as indicated by the name [142]. They function through repressing expression of the homeobox gene. Later biochemical studies reveal PcG proteins function through modifying histones and regulating chromatin status. In drosophila, OGT coding gene, sxc (Super Sex Combs, sxc) is a polycomb gene itself. Consistently, robust O-GlcNAcylation can be detected on chromatin [143, 144]. Notably, PcG proteins form two conserved protein complexes in drosophila and human, called PRC1 and PRC2. PRC1 catalyzes histone H2A-K119 ubiquitination while PRC2 catalyzes histone H3K27 methylation. Both marks are repressive and reversible. Protein complex PR-DUB containing deubiquitinase BAP1 can remove H2A-K119 ubiquitination. PRC1 and PRC2 play critical roles in stem cell fate determination, embryonic development, and cancer [145].

Consistent with OGT’s function as a polycomb protein, proteomic analyses showOGT interactome in HeLa cells containslots of PcG proteins belonging to PRC1, PRC2 andPR-DUB: EZH2, EED, SUZ12, RNF2 (also called RING1B), CBX2, PCGF1, BMI1, BAP1, and ASXL1 [146, 147]. Consistently, OGT/OGA can directly regulate gene expression. O-GlcNAcylation and suppressive histone modifications are co-localized on genes, suppressing gene expression [148, 149]. Conversely, OGA is shown to colocalize to target genes with P300/CBP and activate gene expression [149]. However, the effect of OGT/OGA on transcription is likely context-dependent and gene-specific. For example, OGA can interact with co-repressor mSIN3A/HDAC and repress gene expression as well [150]. Conversely, OGT can interact with gene-activating histone acetyltransferase complex NSL [151]. While it is not impossible some of these physical interaction between OGT/OGA and epigenetic machinery can function without O-GlcNAcylation events, in the next session, we will mainly focus on how OGT/OGA regulates chromatin biology through its O-GlcNAcylation activity.

### O-GlcNAylation of histones

One important advancement towards our understanding of OGT’s effect on chromatin is identifying histones are O-GlcNAcylated [152]. Histone O-GlcNAcylation is now considered as part of histone code.

#### Histone O-GlcNAcylation and mitosis

Initial mass spectrometry identified H2A-T101, H2B-S36, H4-S47 and H3-T32 as potential sites of O-GlcNAcylation [152, 153]. In addition, point mutation suggests H3S10 be O-GlcNAcylated [154]. Overall Histone O-GlcNAcylation increases during recovery from heat shock [152] and is implicated in cell cycle regulation. O-GlcNAc level on histones (particularly H3) decreases during early mitosis and gradually increases during late mitosis to G1 phase. One mechanism of histone O-GlcNAcylation function is through interplay with histone phosphorylation, which is known to be critical for mitosis. Concordantly, OGT overexpression changes the classical mitosis-related histone modifications including H3S10 phosphorylation [153, 155]. Consistently, OGA inhibitor also decreases H3 phosphorylation [153]. Phosphorylation at H3-S10, S28 and S32 is known to be mutually regulated by Aurora B kinase and protein phosphatase 1 (PP1) [156–158]. Interestingly, OGT, OGA, Aurora B and PP1 form a complex in mitosis and localize to midbody, indicating this complex may orchestrate histone o-GlcNAcylation and phosphorylation during mitosis [84].

#### Histone O-GlcNAcylation in DNA damage repair

Histone O-GlcNAcylation can function in other processes other than mitosis. For example, histone O-GlcNAcylation can regulate DNA damage repair. OGT localizes to DNA damage site and GlcNAcylates Histone H2AX-S139, counteracting its phosphorylation at the same site. This mechanism facilitates DNA damage repair by preventing excessive expanding of H2AX-S139 phosphorylation [159]. Accordingly, loss of OGT prolongs G2/M checkpoint and decreases cell viability after DNA damage.

Besides H2AX-S139, OGT also modifies H2A-S20 during DNA damage repair [160]. Among many histone H2A genes in human genome, some H2A isoforms have Serine at position 40. H2A-S40 O-GlcNAcylation increases during DNA damage, which promotes DNA damage repair in association with H2AZ-acetylation or γH2AX. Conversely, H2A-S40A mutant prevents the accumulation of DNA repair apparatus such as DNA-PKcs and Rad51 at the damage site [161].

#### Histone O-GlcNAcylation and gene expression

H2B-S112 O-GlcNAcylation is the most-studied O-GlcNAcylation on histone, which was initially identified by in vitro O-GlcNAcylation coupled with Mass Spectrometry [162]. H2B-S112 O-GlcNAcylation promotes H2B-K120 ubiquitination via two mechanisms. Firstly, H2B-S112 O-GlcNAcylation serves as an anchor for histone H2B ubiquitin ligase RNF20/RNF40 [162]. Secondly, S112-GlcNAcylation increases H2B binding to FACT complex [163]. FACT complex can in turn facilitate the H2B-K120 monoubiquitination by RNF20/40 and UbcH6 [164]. H2B-K120 ubiquitination is a gene-activating modification and consistently S112 O-GlcNAcylation promotes gene transcription [162]. Besides, H2B-S112 O-GlcNAcylation increases at DNA damage foci where it facilitates the recruitment of damage repair proteins [165].

H2A-T101 O-GlcNAcylation, identified with mass spectrometry [152, 162], was later shown to promote an open chromatin state by directly destabilizing H2A/H2B dimer in vitro with chemical biology methodology [166]. Based on this observation, this modification might affect gene expression in vivo.

Above studies have revealed a few different modes by which histone O-GlcNAcylation may function, depending on the modified sites. Firstly, Histone O-GlcNAcylation may prevent or facilitate protein targeting to chromatin. For example H2A-S40 O-GlcNAcylation facilitates the recruitment of DNA-PK and Rad51 to DNA -damage site [161]. Similarly, H2B-S112 modification recruits H2B E3 ligase [162]. Secondly, O-GlcNAcylation of Histone may directly change the nucleosome structure. For example, H2A-S101 may directly destabize H2A-H2B dimer [166]. Thirdly, O-GlcNAcylation may interfere with or directly compete with other modification of histones, especially phosphorylation on the same or adjacent sites. In addition to the O-GlcNAcylated sites discussed above, mass spectrometry has identified other potentially O-GlcNAcylated sites on histones, albeit no function has been ascribed yet. Part of the reason is the study of histone O-GlcNAcylation has been hindered by its low endogenous level in mammalian cells [167].

### O-GlcNAcylation of histone chaperones

Histone deposition into or removal from the nucleosome is facilitated by histone chaperones, which play an important role in chromatin biology. Different histones or histone variants may utilize different chaperones. The histone chaperone HIRA complex deposits histone variant H3.3 to genic regions independent of DNA replication. Increase in H3.3 incorporation into chromatin dependent on HIRA is essential for cancer progression and metastasis [168]. HIRA complex contains histone cell cycle regulator (HIRA), Ubinuclein1 (UBN1), and calcineurin binding protein 1 (CABIN1). UBN1 can interact with OGT and is O-GlcNAcylated at S231 [56]. This modification promotes the integrity of HIRA complex. Accordingly, S231A mutant of UBN1 is defective at depositing H3.3 into genic regions [56].

### O-GlcNAcylation of histone methylation-related proteins

Methylation on Histone lysine or arginine residues is one of the most-studied epigenetic modifications. Methylation of lysine and arginine is catalyzed by two different groups of enzymes respectively with site-specificity. Histone methylation may either positively or negatively affect gene expression, depending on the position and type of the modification. Several histone methyltransferases are shown to be regulated by O-GlcNAcylation.

EZH2, the catalytic subunit of PRC2, catalyzes H3K27 methylation, a repressive mark. EZH2 is implicated in multiple cancer types. OGT can modify human EZH2 at multiple sites [169, 170]. O-GlcNAcylation of N-terminal S73, S76, S84 or T313 promotes EZH2 protein stability while modification at S729 abolishes EZH2 di- and tri-methylation activities but not mono-methylation activity [169, 170]. EZH2 O-GlcNAcylation also occurs in mouse cells. EDAL, a LncRNA, shields mouse EZH2-T309, counterpart of human T313, from O-GlcNAcylation to promote EZH2 lysosomal degradation [171]. Besides, OGT also colocalizes with EZH2 at gene promoters [172]. Consistent with OGT’s effects on EZH2, OGT knockdown decreases cellular H3K27 tri-methylation and re-activates a group of EZH2-targeted tumor suppressor genes in breast cancer cells [169].

H3K79 methylation is another important histone modification, which typically correlates with gene activation. H3K79 methylation is catalyzed by DOT1L [173, 174], which is implicated in both solid cancer and leukemia. Particularly, DOT1L has a well-established role in MLL-fusion leukemia, in which DOT1L is targeted to promoters of genes important for disease initiation and progression, including HOXA9 and MEIS1. Recently, we reported DOT1L could be O-GlcNAcylated [175]. Mass spectrometry, in vitro and in vivo assays show DOT1L is modified at its c-terminal Ser-1511. O-GlcNAcylation is important for DOT1L protein stability. Consistently, glucose starvation decreases DOT1L protein level significantly while HBP metabolites GlcN and GlcNAc can rescue DOT1L protein level. Mechanistically, DOT1L protein has a short half-life and UBE3C functions as its E3 ubiquitin ligase. DOT1L O-GlcNAcylation interferes with its interaction with UBE3C. As a result, O-GlcNAcylated DOT1L is less susceptible to UBE3C-mediated ubiquitination and ensuing degradation. Functionally, O-GlcNAcylation is important for DOT1L protein level in vivo and contributes to overactivation of critical oncogenes in MLL-fusion leukemia [175].

MLL5 belongs to the MLL family H3K4 methyltransferases although itself may not possess such enzymatic activity [176, 177]. MLL5 contains a PHD domain that can recognize H3K4 methylation and contributes to its gene activation function. MLL5 is implicated in different cancer types [176]. MLL5 interacts with OGT and is modified by OGT at Ser-435 and Thr-440. OGT can promote MLL5 protein stability [178]. MLL5 can be recruited by HCF-1 to E2F1-responsive promoters to induce transcriptional activation and cell cycle progression [177]. HCF-1 is a multifunction protein involved in organization of multiple chromatin-modifying complexes including H3K4 methyltransferase complex SET1/COMPASS. HCF-1 also interacts with other histone-modifying enzymes such as demethylase LSD1 [179], Histone acetyltransferase, mSIN3A complex [180] and ATAC histone acetyltransferase complex [181]. Proteolysis of HCF-1 regulates its transcriptional activity. OGT can interact with, O-GlcNAcylate and cleave HCF-1 [117, 182, 183].

CARM1, also known as PRMT4, is a histone arginine methyltransferase. Reported products of CARM1 includes H3R17me2a [184, 185], H3R26me2a [184] and H3R2me2a [184]. CARM1, generally considered a gene activator is a potential therapeutic target in certain cancer types [186]. CARM1 can be O-GlcNAcylated, which affects its substrate specificity [187]. Besides, CARM1 O-GlcNAcylation functions during mitosis as overexpression of OGT prevents mitotic phosphorylationand correct cellular localization of CARM1 during mitosis [155]. Consistently, OGT overexpression changes H3R17 methylation on mitotic chromosome [155].

### O-GlcNAcylation of histone acetylation-related proteins

Histone acetylation is added by several histone acetyltransferase and can be removed by over a dozen histone deacetylases. Dysregulation of these enzymes and histone acetylation is well-documented in cancer. Among histone acetyltransferases, MOF can constitute two different protein complexes, MSL and NSL. NSL3 is a subunit of the NSL acetyltransferase complex. O-GlcNAcylation at Thr-755 inhibits NSL3 polyubiquitination and degradation, consequently enhancing acetylation of histone H4-K5, K8 and K16. NSL3 O-GlcNAcylation contributes to lung cancer cell proliferation [188, 189].

NuRD is a histone deacetylase protein complex, which contains histone deacetylase HDAC1/2 as catalytic subunits. It also contain Mi2-α/β which has helicase and chromatin remodeling activity. NuRD complex has been implicated in various cancer types [190]. A study found Mi2-β interacts with OGT as well as OGA and is O-GlcNAcylated [191]. Inhibiting OGA decreases Mi2-β at target gene promoters, indicating the modification might affect Mi2-β genomic targeting. Yet it is not clear which site is modified or how this modification affects the NuRD complex as well as histone acetylation [191]. MTA1 is another component of NuRD, which bridges NuRD complex and gene-specific transcription factors. MTA1 can be O-GlcNAcylated at serine S237/S241/S246 [192]. O-GlcNAcylation enhanced MTA1 association with the rest of NuRD complex. And promotes genome targeting of NuRD. In breast cancer cells, O-GlcNAcylation of MTA1 promotes the expression of genes involved in adaptation of breast cancer cells to genotoxic stress [192].

SIN3A complex is another histone deacetylase complex with HDAC1 as the catalytic subunit. SIN3A, named after its scaffold subunit, is implicated in cancer [193]. SIN3A and OGT interact with each other, cooperatively repressing transcription in an OGT activity-dependent manner [194]. HDAC1 is o-GlcNAcylated at T114, S145 and S263, which increases its activity [195]. In HCC cells, HDAC1 O-GlcNAcylation promotes P21 transcription repression and cell proliferation [195].

Besides HDAC1/2-containing complexes, OGT/OGA also regulates other HDACs [150]. HDAC4 plays an important role in multiple cancer types [196]. In heart, HDAC4 may be cleaved into two fragments by protease, terminating its HDAC activity. OGT O-GlcNAcylates HDAC4 at Ser-642 which is necessary for the cleavage. Moreover, Ser-642 O-GlcNAcylation interferes with phosphorylation at the same site by CAMKII, which will otherwise promote nuclear localization of HDAC4 and silencing of downstream genes [197].

SIRT1 represents a different type of HDAC which require NAD  +  as a cofactor. OGT can O-GlcNAcylate SIRT1-Ser549, which increases SIRT1 activity. O-GlcNAcylation of SIRT1 increases under genotoxic, oxidative and metabolic stress in cellular and mouse models, which renders protection from stress-induced apoptosis [198]. Besides, OGT can increase SIRT1 protein level in breast cancer [199].

### O-GlcNAcylation of histone ubiquitination-related proteins

H2A-K119 monoubiquitination is a prevalent PTM on nucleosome, which is suppressive for gene expression. H2A-K119 ubiquitination is catalyzed by aforementioned PRC1 complex. PRC1, a RING-finger E3 ligase protein complex, contains RING1 (RING1A or RING1B) as catalytic subunit as well as other components. RING1 is only active when in complex with Bmi-1, another key component in the canonical PRC1 complex [200, 201]. Bmi-1 is overexpressed in multiple cancer types and contributes to tumorigenesis. Recent studies have identified connection between O-GlcNAcylation and PRC1 activity. OGT interacts with Bmi-1 and modifies Bmi-1 at S255, which stabilizes Bmi-1 protein and promotes its oncogenic activity [202]. In addition, OGT can modify RING1B at Thr-250, Ser-251 and Ser-278. O-GlcNAcylation affects RING1B genomic targeting as O-GlcNAcylated and un-modified RING1b are preferentially targeted to different sets of genes in ChIP-seq experiments [203].

H2A-K119 ubiquitination can be removed by deubiquitinases, among which PR-DUB complex is best-studied. PR-DUB contains the catalytic BAP1 and other non-catalytic proteins including ASXL1 [116, 204, 205]. ASXL1 is necessary for gene activation function of PR-DUB [206]. Besides PR-DUB, ASXL1 also forms other protein complexes which can regulate chromatin biology. Functionally, ASXL1 is required to suppress abnormal expression of HOX genes and ASXL1 is frequently mutated in myeloid malignancies [207]. OGT interacts with ASXL1 and modifies ASXL1 at S199, which increases ASXL1 protein stability. Loss of OGT decreases cellular H3K4me3 and H3K27me3. Meanwhile, loss of ASXL1 also changes histone H2B O-GlcNAcylation, indicating ASXL1 and OGT modulate each other’s function [208].

### O-GlcNAcylation of DNA methylation-related proteins

Mammalian genomic DNA can be methylated on the 5-carbon of Cytosine in CpG dinucleotides. DNA methylation plays pivotal roles for chromatin function. For example, DNA methylation on gene promoters is a well-known repressive mark [209]. DNA methylation is catalyzed by a few DNA methyltranferases [210]. On the other hand, TET proteins, including TET1/2/3, can facilitate the removal of 5mC from genome by sequential oxidation of 5mC to 5-hydroxymethyl-Cytidine (5hmC), 5-formyl-Cytidine (5fC) and 5-carboxy-Cytidine (5caC). Besides being intermediators of 5mC removal, 5hmC, 5fC and 5caC may also have functions different from that of 5mC [211]. TET proteins are important in both development and diseases including cancer [211]. Multiple studies have identified functional interplay between TET proteins and OGT. All three TET proteins can interact with OGT, which can promoteeach other’s targeting and activity on chromatin. They together affect other histone modifications [125, 212–216]. Besides, all three TET can be O-GlcNAcylated. TET1 O-GlcNAcylation increases its protein level in embryonic stem cells. Consistently, loss of OGT leads to decreased TET1 protein level and 5hmC on Tet1-target genes [213]. For TET3, O-GlcNAcylation can promote its nuclear export and consequently decrease the formation of 5hmC by TET3 in somatic cells such as 293 T, HeLa and NIH3T3 [215]. Consistently, higher glucose concentration promotes TET3 translocation into the cytoplasm [215].

### O-GlcNAcylation of other chromatin regulators

TRIM28 is a multifunction chromatin-binding protein, which plays an important role in many cancers. TRIM28 interacts with other histone modifiers and regulates chromatin biology. One established function of TRIM28 is silencing the endogenous retrovirus elements by recruiting other epigenetic machinery [217, 218], which is associated with cancer [219–221]. OGT interacts with TRIM28 and GlcNAcylates TRIM28-bound proteins. The activity of OGT is important for TRIM28-mediated retrovirus silencing. Consistently, targeting OGA to retroelement promoters leads to retrovirus activation [222].

The above studies have shown O-GlcNAcylation can regulate epigenetic machinery directly or indirectly. O-GlcNAcylation may have direct effects on epigenetic machinery, including changes in protein stability, activity, substrate preference, protein–protein interaction/complex assembly, genomic targeting and subcellular localization. Besides, O-GlcNAcylation of other proteins may regulate epigenetic machinery and chromatin modifications indirectly.

### Potential mechanisms of differential O-GlcNAcylation of epigenetic machinery

OGT modifies and regulates many substrates involved in epigenetic modifications, some of which even seemingly counteract each other’s function. So a compelling question is how OGT, a single enzyme, can orchestrate so many downstream epigenetic changes It is reasonable to propose that OGT regulates these substrates differentially. For example when a target gene needs to be silenced, the cell needs to inactivate or preclude the activating machinery while activating or engaging the suppressing machinery. Changes in HBP pathway, OGT expression or activity en bloc are not enough to realize such differential regulation. As indicated by the studies discussed above, multiple additional mechanisms may contribute. Firstly, multi-valent protein–protein interaction likely play a major role in differential regulation. OGT might only modify a substrate when it is in an appropriate protein complex wherein multivalent interaction between OGT and that complex renders the O-GlcNAcylation efficient. Indeed, as shown in many cases, OGT can interact with more than one components in a protein complex. Conversely, OGT activity on a substrate may be obstructed by other proteins interacting with OGT or the substrate. Secondly, OGT action on a substrate may be prevented or facilitated by a pre-existing modification on the substrate or OGT. This pre-existing modification could be differentially regulated by oncogenic signals. Thirdly, OGT activity on a substrate might be tightly regulated by OGT compartmentation. Pertinent to this review, many OGT substrates are chromatin-bound including histones while OGT interaction with chromatin is subject to multiple regulations. Besides, OGT targets to sub-genomic regions differently. Such precise targeting of OGT is regulated by OGT nuclear localization, interacting-proteins and local chromatin environments. For example, TET2/3 interacts with OGT and facilitates OGT recruitment to histone, leading to increased H2B-S112-GlcNAc [125].

For each of these three mechanisms, the role of upstream signaling pathways cannot be over-emphasized. Upstream signals may regulate the multi-valent interaction between OGT and substrate-containing protein complex. These signals may modify OGT which either facilitates or interferes with its interaction with a potential substrate. In addition, the formation and architecture of these multi-component protein complexes could be regulated by cancer-related signals, which may affect the O-GlcNAcylation. Upstream signals may also directly modify epigenetic machinery which facilitates or interferes with O-GlcNAcylation. For example, phosphorylation may compete for the same site with O-GlcNAcylation [225]. In addition, upstream signals can directly regulate the subcellular localization of OGT and/or its potential substrates, which will affect the O-GlcNAcylation [127, 128]. To take an example, the integrity of PRC2 complex is under the regulation of AMPK [226]. Through phosphorylating EZH2, AMPK can decrease cellular H3K27 methylation. Meanwhile, AMPK can phosphorylate OGT, which changes its chromatin targeting and proteomic substrate spectrum [61, 62]. OGT modifies EZH2 on multiple sites and regulates its protein stability as well as enzymatic activity. Collectively, AMPK may regulate EZH2 O-GlcNAcylation through both OGT phosphorylation and EZH2 phosphorylation. Consequently, AMPK might regulate EZH2 O-GlcNAcylation both globally and sub-genome specifically. Studies have identified other direct effects of oncogenic signals on epigenetic machinery as well. So it can be expected oncogenic signals not only regulate the O-GlcNAcylation landscape through their effects on HBP enzymes and OGT/OGA but also differentially fine-tune each O-GlcNAcylation event on the epigenetic machinery spatially and temporally.

## Conclusion and perspective

**Fig. 2:**
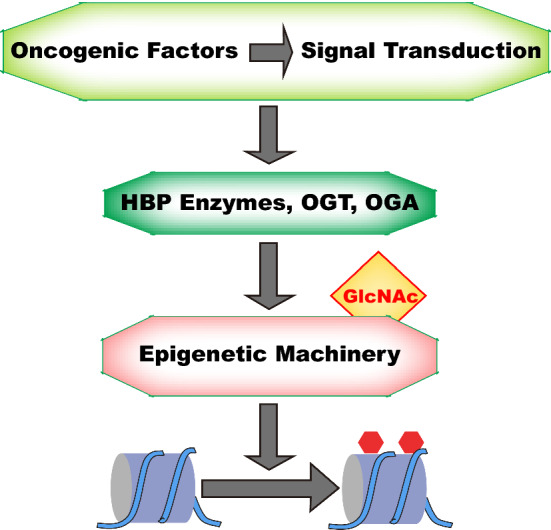
Oncogenic signal/O-GlcNAcylation/Epigenetic Modification Axis. Virus Oncoproteins and Tumor microenvironmental factor can regulate HBP enzymes and OGT/OGA directly or indirectly through intracellular oncogenic signals. Consequently, abnormality in HBP enzymes/OGT/OGA can cause abnormality in epigenetic modification in cancer

Understanding how oncogenic signals change cancer epigenetics is instrumental for cancer etiology. More and more epigenetic machinery are identified to be regulated by O-GlcNAcylation. Our understanding on epigenetic machinery is still fast-growing while new epigenetic modifications, new readers, new writers and new erasers are being identified. We have reason to believe more interplay between O-GlcNAcylation and epigenetic machinery will be identified. For example, proteomic studies are identifying more epigenetic regulators to be O-GlcNAcylated [227]. On the other hand, oncogenic signals have extensive impact on cellular O-GlcNAcylation through regulating HBP enzymes and OGT/OGA. It is reasonable to postulate oncogenic signals can cause the abnormality in epigenetic modification in cancer through O-GlcNAcylation of epigenetic machinery (Fig. 2). Furthermore, these oncogenic signals might modulate the O-GlcNAcylation of each epigenetic machinery in a time- and space-specific manner. Future study shall reveal more direct evidences for these aspects. The knowledge of oncogenic signal/O-GlcNAcylation/epigenetics axis (Fig. 2) will broaden and deepen our understanding of cancer. Abnormality of O-GlcNAcylation in cancer is wide-spread. As OGT knockdown or inhibition has shown anti-tumor effects in vitro and in mouse models, OGT has been suggested as a potential therapy target [10]. Yet so far there is no OGT inhibitor suitable for clinical testing. So it is not known whether we can directly target OGT for cancer treatment [10]. Nevertheless as our understanding towards the upstream regulators and downstream effectors of O-GlcNAcylation grows, we shall be able to target its regulation and function in cancer.

## Data Availability

Not applicable.
